# Phenol-Rich Botanicals Modulate Oxidative Stress and Epithelial Integrity in Intestinal Epithelial Cells

**DOI:** 10.3390/ani12172188

**Published:** 2022-08-25

**Authors:** Andrea Toschi, Andrea Piva, Ester Grilli

**Affiliations:** 1 Vetagro S.p.A., Via Porro 2, 42124 Reggio Emilia, Italy; 2 Department of Veterinary Medical Sciences, University of Bologna, Via Tolara di Sopra n. 50, 40064 Ozzano dell’Emilia, Italy; 3 Vetagro, Inc., 17 East Monroe Street Suite #179, Chicago, IL 60604, USA

**Keywords:** Caco-2 cells, intestinal function, antioxidant, phenol-rich compounds, polyphenols, botanicals

## Abstract

**Simple Summary:**

Phenolic compounds are one of the largest groups of botanical components able to provide advantageous effects. Phenol-rich botanicals are widely investigated for their numerous characteristics, mainly antioxidant and anti-inflammatory. Despite the increasing use of these substances in food and feed, their effect on intestinal mucosa is still marginally known. The aim of this study was to screen the activity of different sources of phenol compounds on intestinal oxidation and barrier integrity in vitro. Human intestinal cells were treated with increasing doses of phenol-rich botanicals, then, an evaluation of oxidative stress and mucosal integrity markers was performed. Different sources of phenolic compounds elicit different responses from an organism, although all the tested botanicals showed a positive effect on treated cells. The results suggest that the potential use of these botanicals as dietary supplements could improve intestinal barrier function.

**Abstract:**

Botanicals are mainly known for their role as antimicrobials and anti-inflammatories. Thus, the dual purpose of the study was to verify the antioxidant potential of the tested botanicals and to evaluate their possible modulation of intestinal barrier integrity. As the effects of various phenol-rich extracts were screened, the human Caco-2 cell line was determined to be most suitable for use as the in vitro model for the intestinal epithelium. The tested botanicals, all approved as feed additives, are ginger essential oil, tea tree oil, grape seed extract, green tea extract, olive extract, chestnut extract, pomegranate extract, thyme essential oil, and capsicum oleoresin. The cells were treated with incremental doses of each botanical, followed by measurements of transepithelial electrical resistance (TEER), gene expression of tight junctions (TJs), and reactive oxygen species (ROS). The results showed how different phenol-rich botanicals could modulate barrier functions and oxidative stress in different ways. Interestingly, all the botanicals tested exerted an antioxidant potential by dropping the cytoplasmatic ROS, while the beneficial effect was exerted at different concentrations for each botanical. Our data support the role of plant extracts and essential oils in controlling gut barrier function and in reducing the negative effects of oxidative stress in intestinal epithelial cells, thereby supporting gut barrier functionality.

## 1. Introduction

Botanicals are complex compounds constituted of aromatic plants’ secondary metabolites, whose antibacterial and insecticide functions play a central role in the defense of the plants [1]. Known for their antiseptic properties and their aromatic profile, they are used as food preservatives, antimicrobials, analgesics, sedatives, anti-inflammatories, and local anesthetic remedies [1]. In relation to the process of extraction, they could be powder extracts, essential oils (EOs), or oleoresins (ORs). The composition of botanicals is highly variable, since they depend on the plant species, the stage of development, the chemotype, and the part used for the extraction, as well as environmental temperature, humidity, or the composition of the soil [2]. Botanicals are characterized by 2–3 major components with a high concentration (up to 70%), among a total of 20–60 components [3]. In terms of effects and biological activity, each chemical constituent has its own characteristic properties. Over the past few years, the number of studies conducted to investigate the benefits of botanicals, both for animal production and for human health and nutrition, has considerably increased [4,5,6]. In animals, the use of natural products seems convenient not only to increase the voluntary feed intake, but also to aid with nutrient absorption and to support the immune system [7]. Moreover, botanicals appear to be able to promote intestinal health by improving intestinal barrier function, which clearly plays a key role in animal health and productivity [8,9].

Phenolic compounds are one of the largest groups of botanical components able to provide advantageous effects. In particular, polyphenols are natural compounds that normally occur in a large variety of plants, with several biological activities. Botanical components belonging to the class of polyphenols are flavonoids, anthocyanidins, resveratrol, tannins, and phenolic acids [10]. Polyphenols and flavonoids are largely known for their ability to interact with ROS (reactive oxygen species), modulating the redox balance and preventing cell damage [11]. As farm animals are particularly susceptible to oxidative stress, which leads to tissue damage and lipoperoxidation of the final product, the addition of polyphenols as feed additives in their diet is growing in popularity [12]. Besides their antioxidant capacity, botanicals rich in phenols are also known as antibacterial, anti-inflammatory, antiseptic, antiparasitic, and immunomodulatory [11,13,14]. As in animal and human nutrition, there are several sources of phenolic compounds, in this study, we screened different botanicals, all approved as feed additives: ginger essential oil (GEO), tea tree oil (TTO), grape seed extract (GSE), green tea extract (GTE), olive extract (OE), pomegranate extract (POM), chestnut extract (CHE), thyme essential oil (TEO), and capsicum oleoresin (CAP).

The aim of this study was to evaluate the potential role of phenol-rich botanicals in improving intestinal health, in terms of antioxidant capacity and barrier integrity. As various botanicals were screened, it was decided to use an in vitro approach. The human epithelial cell line Caco-2 was used to verify the effects on intestinal epithelium integrity and oxidative status.

## 2. Materials and Methods

### 2.1. Chemicals and Reagents

Chemicals and cell culture reagents were provided by Merck Life Science S.r.l. (Milan, Italy), except where otherwise specified. GEO and TTO were purchased from Frey&Lau (Frey + Lau GmbH, Henstedt-Ulzburg, Germany), GSE, GTE, and OE were obtained from Layn Natural Ingredients (Guilin Layn Natural Ingredients Corp., Shanghai, China), POM and CHE were acquired from Layn Corp (Layn Europe SRL., Savona, Italy), and CAP and TEO were obtained from Frey&Lau (Frey + Lau GmbH, Henstedt-Ulzburg, Germany). Stock solutions of all the botanicals were prepared in ethanol up to 100% (*v/v*) to ensure their solubility, and supplemented in culture medium with a final concentration of ethanol ≤0.5% (*v/v*).

### 2.2. Intestinal Caco-2 Culture Conditions

The human colon adenocarcinoma cell line (Caco-2) was obtained from DSMZ (DSMZ-German Collection of Microorganisms and Cell Cultures, Leibniz Institute, Braunschweig, Germany). Caco-2 cells were maintained at 37 °C in an atmosphere containing 5% CO_2_ at 95% relative humidity, in a basal medium composed by DMEM supplemented with 10% fetal bovine serum (FBS), 1% L-Glutamine, 1% penicillin/streptomycin (P/S), and 1% non-essential amino acids (NEAA). Caco-2 cells were cultured in two different supports depending on the subsequent analysis: (1) 96-well plates, seeded at a density of 1.5 × 10^4^ cells/well, to evaluate viability and oxidative response; (2) 24-well transwell polyethylene inserts (0.4 µm pore; Corning, Amsterdam, Nederland), seeded at a density of 5 × 10^4^ cells/transwell, to evaluate electrophysiological parameters. For all the following analyses, control groups were incubated in medium containing ≤0.4% (*v/v*) of ethanol (corresponding to the highest concentration of ethanol present in culture because of the stock in ethanol of botanicals) to exclude any possible cytotoxic effects mediated by ethanol.

### 2.3. Viability Assay on Caco-2

Cell viability to all the botanicals tested was evaluated with PrestoBlue™ reagent (Invitrogen, Thermofisher Scientific, Milan, Italy). Caco-2 cells seeded onto 96-well plates were treated with specific substances and their relative control. Concentrations of the substances were selected to meet and exceed the suggestion of the producer for the use of these compounds as feed additives; these concentrations were: 1, 10, 100, and 200 ppm. Cells were tested for their viability both 24 h and 7 days after each treatment. Fluorescence values were recorded with a Varioskan™ LUX (Thermofisher Scientific, Milan, Italy). The percentage of cell viability was calculated using the following formula [15]:

Cell Viability % = Mean Fluo/Mean control × 100(1)

### 2.4. Measurement of Reactive Oxygen Species (ROS) Level

ROS were measured using CellROX^®^ Deep Red Reagent (Thermofisher Scientific, Milan, Italy), following the manufacturer’s instructions. Briefly, CellROX^®^ Deep Red Reagent is a fluorogenic probe meant to measure cellular oxidative stress in living cells, in particular, the signal is localized in the cytoplasm. The cell-permeable reagent is non-fluorescent while in a reduced state, whereas it becomes fluorescent upon oxidation by reactive oxygen species, with emission maxima ~665 nm. Caco-2 cells seeded onto 96-well plates were treated with 1, 10, and 100 ppm of botanicals for 24 h. CellROX^®^ Deep Red Reagent was added at a final concentration of 5 μM to the cells and then incubated for 30 min at 37 °C. Subsequently, the medium was removed, and the cells were washed three times with DPBS (Dulbecco’s phosphate buffered saline). Fluorescence data were measured with a Varioskan™ LUX (Thermofisher Scientific, Milan, Italy). Vitamin E and vitamin C were used as a standard of antioxidant potential, cells were treated for 24 h as for botanicals. Hydrogen peroxide 500 μM was added 60 min before treatment with CellROX^®^ as the ROS-inducer molecule.

### 2.5. Dose Response on Caco-2 Cells

Twenty-eight days post-seeding on filters, the experiment started, when Caco-2 cells showed a TEER (transepithelial electrical resistance) value > 600 Ω cm^2^, which indicated adequate monolayer integrity [16]. Cells on transwell filters were distributed into groups and treated with different doses of the botanicals, tested for 7 days. The range of concentrations to be tested was selected considering the inclusion of these botanicals suggested by the producer for their use as feed additives. For this reason, all the substances were tested at 10 and 100 ppm. During the dose response, TEER was measured at 0, 2, 5, and 7 days, using an epithelial tissue voltohmmeter (Millicell ERS-2, Merk, Merckmillipore, Darmstadt, Germany). The data were presented as a percentage of initial value of TEER before the treatments.

### 2.6. Gene Expression Analysis

At the end of the dose-response experiments, cells were collected and stored at −80 °C in a lysis buffer (Macherey-Nagel, Düren, Germany) until gene expression analysis. According to the manufacturer’s instruction, RNA was isolated using a NucleoSpin RNA Kit (Macherey-Nagel, Düren, Germany), and genomic DNA contamination was removed with deoxyribonuclease (rDNA RNase-Free; Macherey-Nagel, Düren, Germany). Measurements at A260/A280 were used to evaluate RNA yield and quality (Varioskan™ LUX, Thermofisher Scientific, Milan, Italy). A total of 500 ng of RNA was reverse-transcribed for each sample with an iScript cDNA Synthesis Kit (Bio-Rad Laboratories Inc., Hercules, CA, USA). Real-time PCR was performed using a CFX Connect Real-Time PCR Detection System and iTaq Universal SYBR Green Supermix (Bio-Rad). Gene expression was normalized using two reference genes: glyceraldehyde-3-phosphate dehydrogenase (GAPDH) and ribosomal protein lateral stalk subunit P0 (RPLP0) [15]. A modification of the 2^−^^ΔΔCT^ method [17] was used to analyze the relative expression (fold changes), calculated relative to the control group. The primer sequence, expected product length, accession number in the GenBank database, and reference are shown in Table 1. Primers were obtained from Merck Life Science S.r.l.

### 2.7. Statistical Analysis

GraphPad Prism^®^ (GraphPad Software, Inc., La Jolla, CA, USA) was used to analyze data. ANOVA repeated measures were used for TEER. One-way ANOVA followed by Bonferroni and Tukey’s post-test was used for the PrestoBlue™ reagent, CellRox^®^ Deep Red assay, and gene expression analysis. The experimental unit was the well (*n* = 6). Differences were considered significant at *p* < 0.05.

## 3. Results

### 3.1. Ginger Essential Oil

#### 3.1.1. Viability and Dose Response

**Figure 1:**
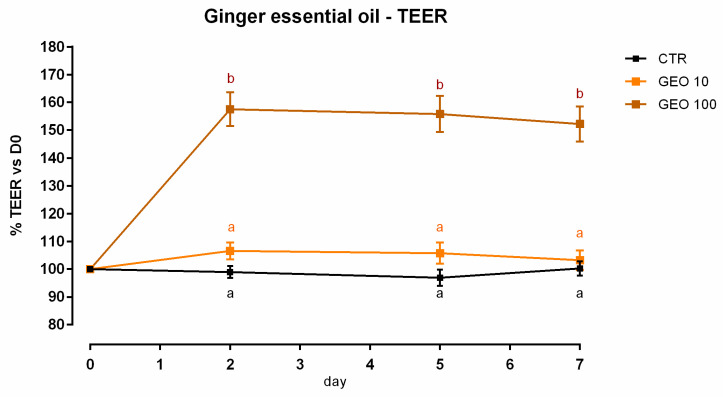
TEER of Caco-2 cells cultured with 10 or 100 ppm of ginger essential oil. Data in the graph are represented as means (*n* = 6) ± SEM. Different letters indicate statistical significance with *p* < 0.05. GEO = ginger essential oil, TEER = transepithelial electrical resistance (Ω·cm^2^).

**Figure 2:**
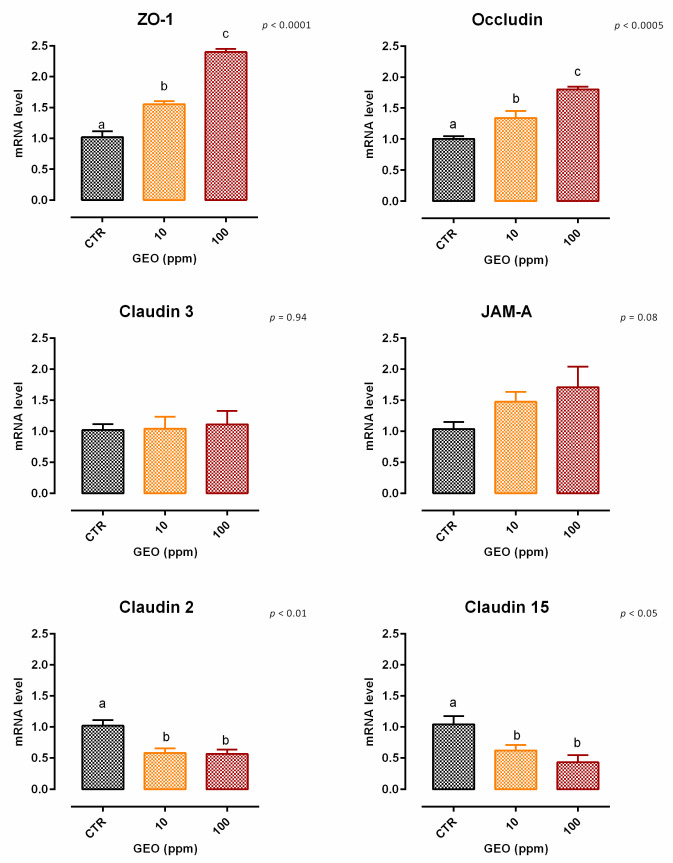
mRNA expression of tight junctions after 7 days of treatment with ginger essential oil. Data are least square means (*n* = 6) ± SEM. Different letters indicate statistical significance with *p* < 0.05. ZO-1 = zonula occludens 1, JAM-A = Junctional adhesion molecules A.

A slight but significant reduction (about 9%) in viability was reported with 100 ppm of GEO after 7 days of treatment (Figure S1). Concerning TEER, 100 ppm of GEO significantly increased the electrical resistance starting from day 2, and the increment remained significant during the entire study, compared to the control and the other inclusion (Figure 1). Results regarding gene expression are consistent with TEER, as reported in Figure 2. Compared to the control, mRNA levels of ZO-1 and occludin were significantly improved by the treatments in a dose-response manner (*p* < 0.0005). Additionally, JAM-A was tendentially increased by the treatments (*p* = 0.08). Conversely, both claudin 2 and claudin 15 mRNA levels were significantly reduced by the addition of GEO, with a stronger reduction with 100 ppm of inclusion (*p* < 0.05). No differences were reported for claudin 3.

#### 3.1.2. Antioxidant Activity

**Figure 3:**
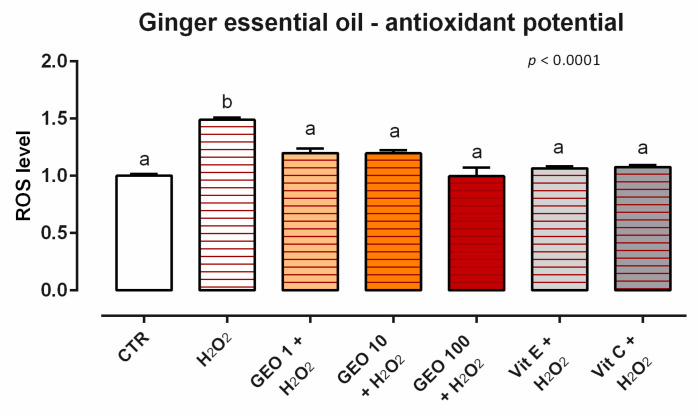
Effects of ginger essential oil on intracellular ROS production: ROS level of Caco-2 cells treated with 1, 10, or 100 ppm of GEO and challenged with 500 μM of hydrogen peroxide (H_2_O_2_) for 60 min. ROS production was assessed by CellROX^®^ deep red reagent assay and expressed as fluorescence intensity calculated relative to the control group. Values in the graph are means (*n* = 6) ± SEM. Different letters indicate statistical significance with *p* < 0.05. 500 μM of hydrogen peroxide (H_2_O_2_) was used as a positive control. Vitamin E (Vit E) and vitamin C (Vit C) were used as a standard of antioxidant potential. ROS = Reactive Oxygen Species, GEO = ginger essential oil.

After 24 h of incubation, GEO did not induce ROS production for any of the concentrations tested (data not shown). Moreover, when cells were challenged with H_2_O_2_ for 60 min (ROS-production challenge, positive control), all of the treatments with GEO allowed for a significant reduction of ROS concentration compared to the positive control, maintaining the same level of ROS as the unchallenged group and vitamins (internal controls) (Figure 3).

### 3.2. Tea Tree Oil

#### 3.2.1. Viability and Dose Response

**Figure 4:**
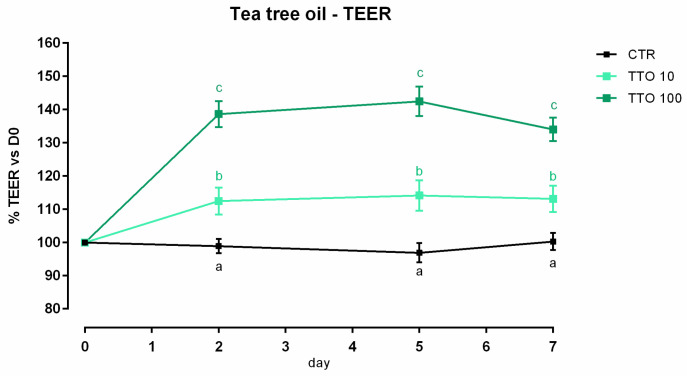
TEER of Caco-2 cells cultured with 10 or 100 ppm of tea tree oil. Data in the graph are represented as means (*n* = 6) ± SEM. Different letters indicate statistical significance with *p* < 0.05. TEER = transepithelial electrical resistance (Ω·cm^2^).

**Figure 5:**
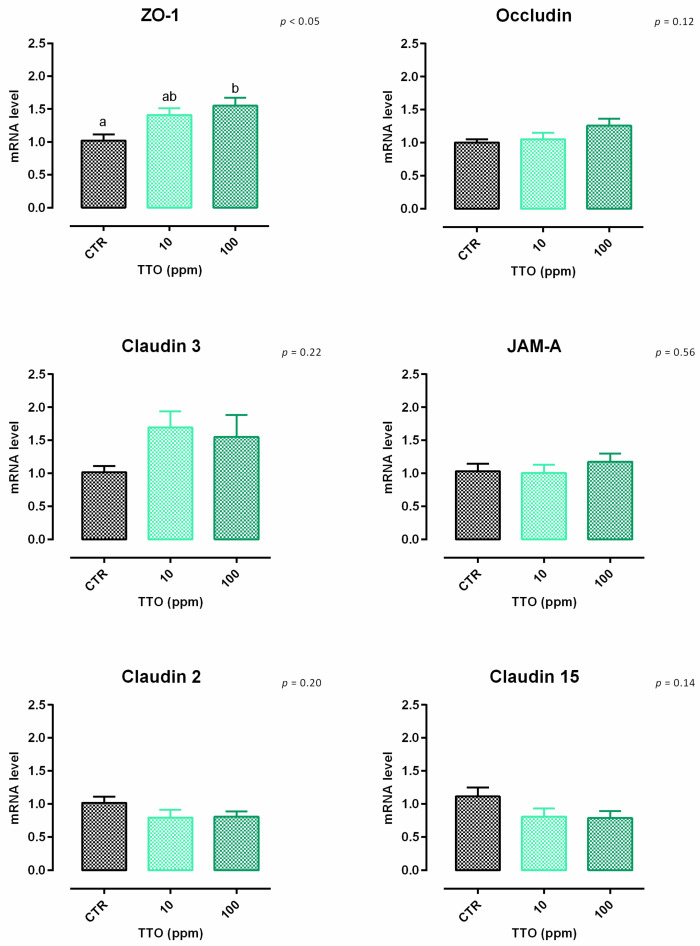
mRNA expression of tight junctions after 7 days of treatment with tea tree oil. Data are least square means (*n* = 6) ± SEM. Different letters indicate statistical significance with *p* < 0.05. ZO-1 = zonula occludens 1, JAM-A = Junctional adhesion molecules A.

After 7 days, a significant reduction of viability was reported, starting from 200 ppm of inclusion (Figure S2). Concerning the dose response, both 10 and 100 ppm of TTO positively impacted the TEER immediately after the start of the treatment, and the increment remained constant throughout the entire study in a dose-dependent manner, compared to the control (Figure 4). Results regarding gene expression are reported in Figure 5. Compared to the control, the mRNA level of ZO-1 was significantly enhanced by the supplementation of 100 ppm of TTO (*p* < 0.05). No differences were reported for occludin, JAM-A, claudin 2, claudin 3, or claudin 15.

#### 3.2.2. Antioxidant Activity

**Figure 6:**
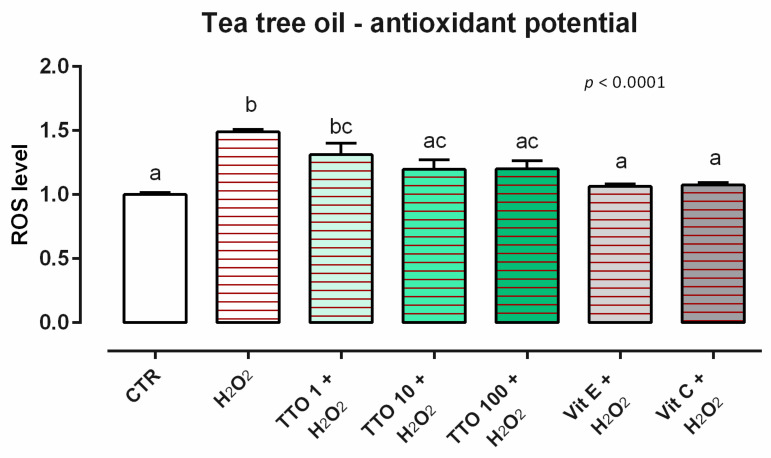
Effects of tea tree oil on intracellular ROS production: ROS level of Caco-2 cells treated with 1, 10, or 100 ppm of TTO and challenged with 500 μM of hydrogen peroxide (H_2_O_2_) for 60 min. ROS production was assessed by CellROX^®^ deep red reagent assay and expressed as fluorescence intensity calculated relative to the control group. Values in the graph are means (*n* = 6) ± SEM. Different letters indicate statistical significance with *p* < 0.05. 500 μM of hydrogen peroxide (H_2_O_2_) was used as a positive control. Vitamin E (Vit E) and vitamin C (Vit C) were used as a standard of antioxidant potential.

As concerns the ROS levels, 24 h of incubation with TTO induces ROS production in Caco-2 cells at the tested concentrations (data not shown). When cells were subjected to a challenge with 500 µM of H_2_O_2_, the addition of TTO positively impacted the ROS production, with a reduction of cells treated with 10 and 100 ppm of TTO compared to the positive control. The reduction of ROS obtained with TTO was comparable to the ones obtained with vitamins, which are used as internal controls (Figure 6).

### 3.3. Grape Seed Extract

#### 3.3.1. Viability and Dose Response

**Figure 7:**
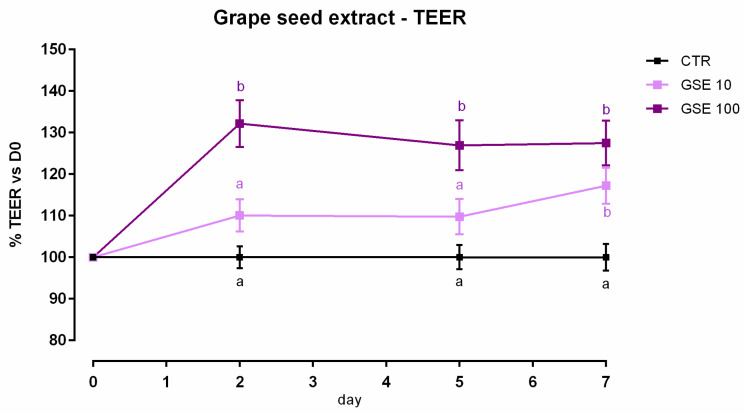
TEER of Caco-2 cells cultured with 10 or 100 ppm of grape seed extract. Data in the graph are represented as means (*n* = 6) ± SEM. Different letters indicate statistical significance with *p* < 0.05. TEER = transepithelial electrical resistance (Ω·cm^2^).

**Figure 8:**
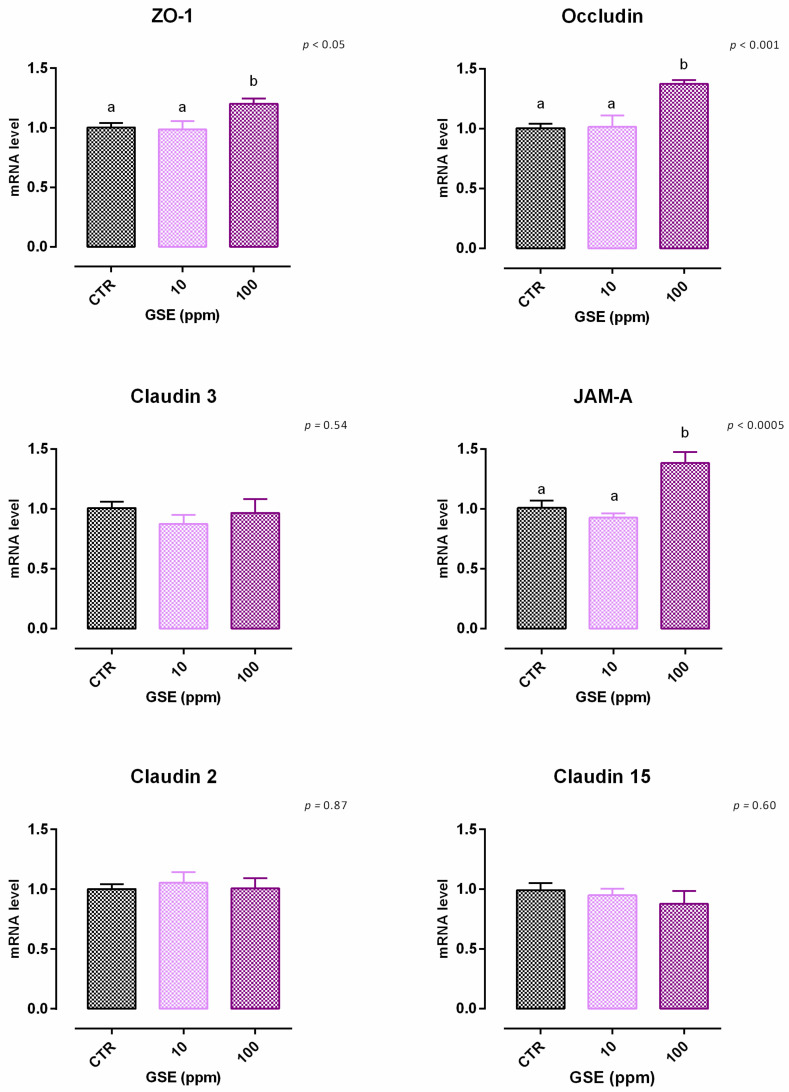
mRNA expression of tight junctions after 7 days of treatment with grape seed extract. Data are least square means (*n* = 6) ± SEM. Different letters indicate statistical significance with *p* < 0.05. ZO-1 = zonula occludens 1, JAM-A = Junctional adhesion molecules A.

Data obtained from the viability assay reported a significant reduction after 24 h of treatment with 200 ppm of extract. Data were confirmed after 7 days, as 100 ppm caused a 10% reduction of viability (Figure S3). Concerning TEER, cells treated with GSE 100 ppm showed an increase of TEER compared to the control, starting from day 2 (Figure 7). Moreover, the addition of 10 ppm revealed an increase of TEER on day 7. Results of gene expression are described in Figure 8. Compared to the control, mRNA level of ZO-1, occludin, and JAM-A were significantly increased by the treatments (*p* < 0.05, *p* < 0.001, and *p* < 0.0005, respectively). No differences were reported for claudins.

#### 3.3.2. Antioxidant Activity

**Figure 9:**
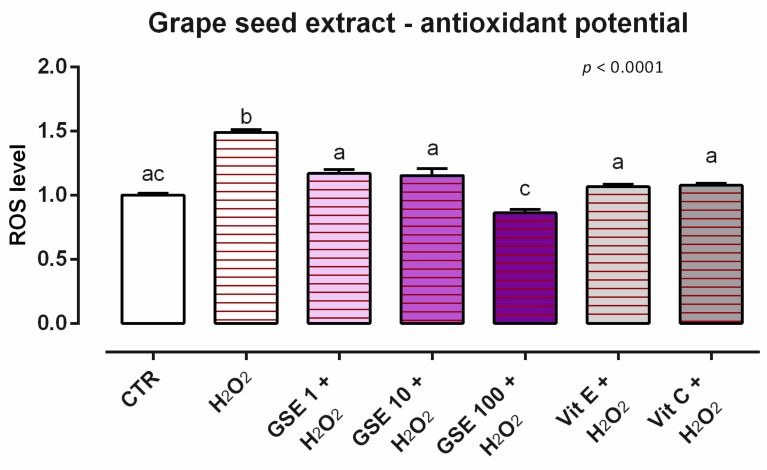
Effects of grape seed extract on intracellular ROS production: ROS level of Caco-2 cells treated with 1, 10, or 100 ppm of GSE and challenged with 500 μM of hydrogen peroxide (H_2_O_2_) for 60 min. ROS production was assessed by CellROX^®^ deep red reagent assay and expressed as fluorescence intensity calculated relative to the control group. Values in the graph are means (*n* = 6) ± SEM. Different letters indicate statistical significance with *p* < 0.05. 500 μM of hydrogen peroxide (H_2_O_2_) was used as a positive control. Vitamin E (Vit E) and vitamin C (Vit C) were used as a standard of antioxidant potential.

After 24 h of incubation with the different dosages of GSE (1, 10, 100 ppm), no variations in ROS productions were reported (data not shown). Moreover, when cells were challenged with H_2_O_2_ for 60 min, the preventive treatment with 1 ppm of GSE significantly ameliorate the amounts of ROS compared to the positive control, maintaining the same level of ROS as the unchallenged group (Figure 9). In fact, all of the doses of GSE allowed a restorative effect against the challenge of the ROS-inducer, with results comparable to vitamin C and vitamin E.

### 3.4. Green Tea Extract

#### 3.4.1. Viability and Dose Response

**Figure 10:**
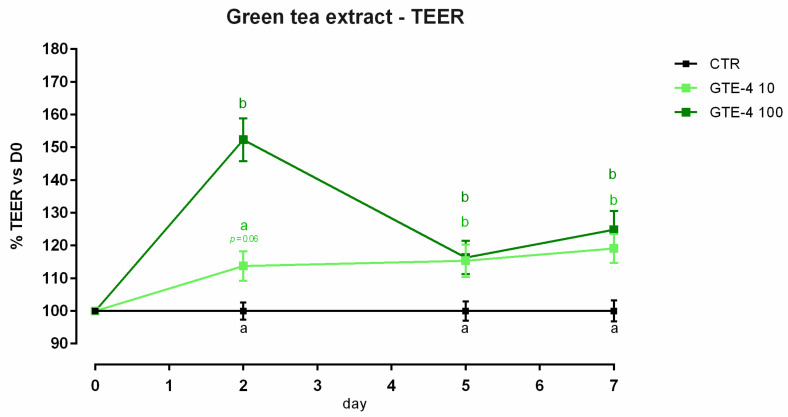
TEER of Caco-2 cells cultured with 10 or 100 ppm of green tea extract. Data in the graph are represented as as means (*n* = 6) ± SEM. Different letters indicate statistical significance with *p* < 0.05. TEER = transepithelial electrical resistance (Ω·cm^2^).

**Figure 11:**
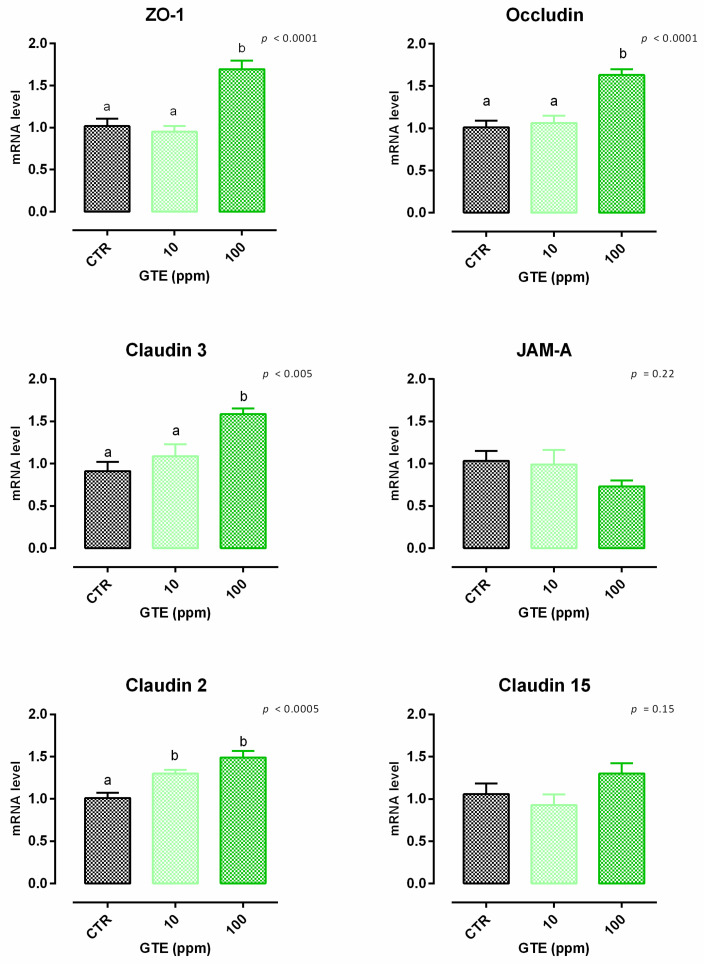
mRNA expression of tight junctions after 7 days of treatment with green tea extract. Data are least square means (*n* = 6) ± SEM. Different letters indicate statistical significance with *p* < 0.05. ZO-1 = zonula occludens 1, JAM-A = Junctional adhesion molecules A.

No significant variation of viability was reported in cells treated with GTE (Figure S4). Concerning TEER, cells treated with GTE 100 ppm showed a peak of increment in TEER compared to the control, starting from day 2 (Figure 10). Treatment with 10 ppm of GTE allows a slow increase of TEER, with a tendency at day 2 (*p* = 0.06 compared to the control); this increment becomes comparable to GTE 100 on days 5 and 7. Results regarding gene expression are reported in Figure 11. Compared to the control, mRNA levels of ZO-1, occludin, claudin 2, and claudin 3 were significantly increased by the treatments (*p* < 0.005). No differences were reported for claudin 15 and JAM-A.

#### 3.4.2. Antioxidant Activity

**Figure 12:**
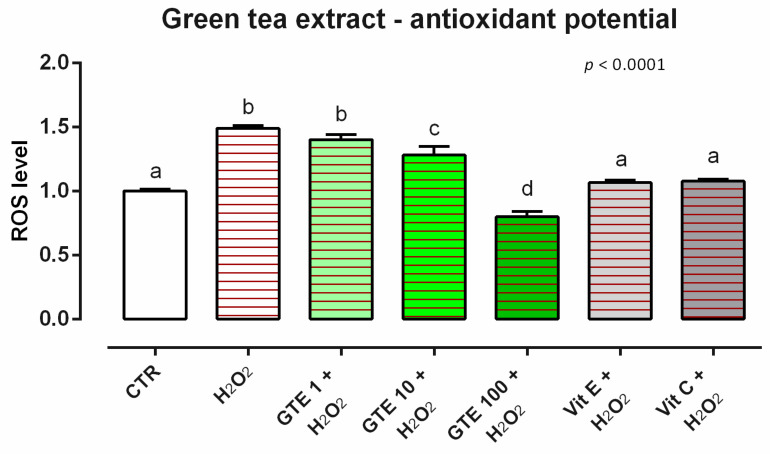
Effects of green tea extract on intracellular ROS production: ROS level of Caco-2 cells treated with 1, 10, or 100 ppm of GTE and challenged with 500 μM of hydrogen peroxide (H_2_O_2_) for 60 min. ROS production was assessed by CellROX^®^ deep red reagent assay and expressed as fluorescence intensity calculated relative to the control group. Values in the graph are means (*n* = 6) ± SEM. Different letters indicate statistical significance with *p* < 0.05. 500 μM of hydrogen peroxide (H_2_O_2_) was used as a positive control. Vitamin E (Vit E) and vitamin C (Vit C) were used as a standard of antioxidant potential.

Concerning the ROS levels, 24 h of incubation with GTE induced ROS production in Caco-2 cells at the tested concentrations (data not shown). When cells were subjected to a challenge with 500 µM of H_2_O_2_, the addition of GTE positively impacted the ROS production, with a reduction of cells treated with 10 and 100 ppm of TTO. In particular, an intermediate reduction of ROS was visible with 10 ppm, while the preventive treatment with GTE 100 ppm allowed a complete reduction of ROS, with values lower than control and vitamins (Figure 12).

### 3.5. Olive Extract

#### 3.5.1. Viability and Dose Response

**Figure 13:**
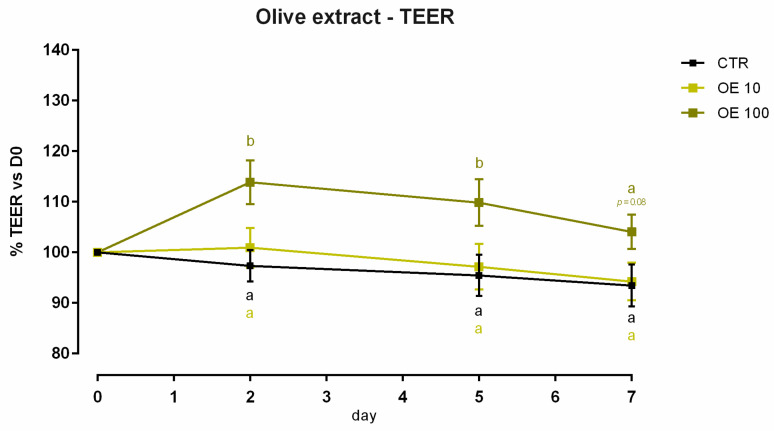
TEER of Caco-2 cells cultured with 10 or 100 ppm of olive extract. Data in the graph are represented as means (*n* = 6) ± SEM. Different letters indicate statistical significance with *p* < 0.05. TEER = transepithelial electrical resistance (Ω·cm^2^).

**Figure 14:**
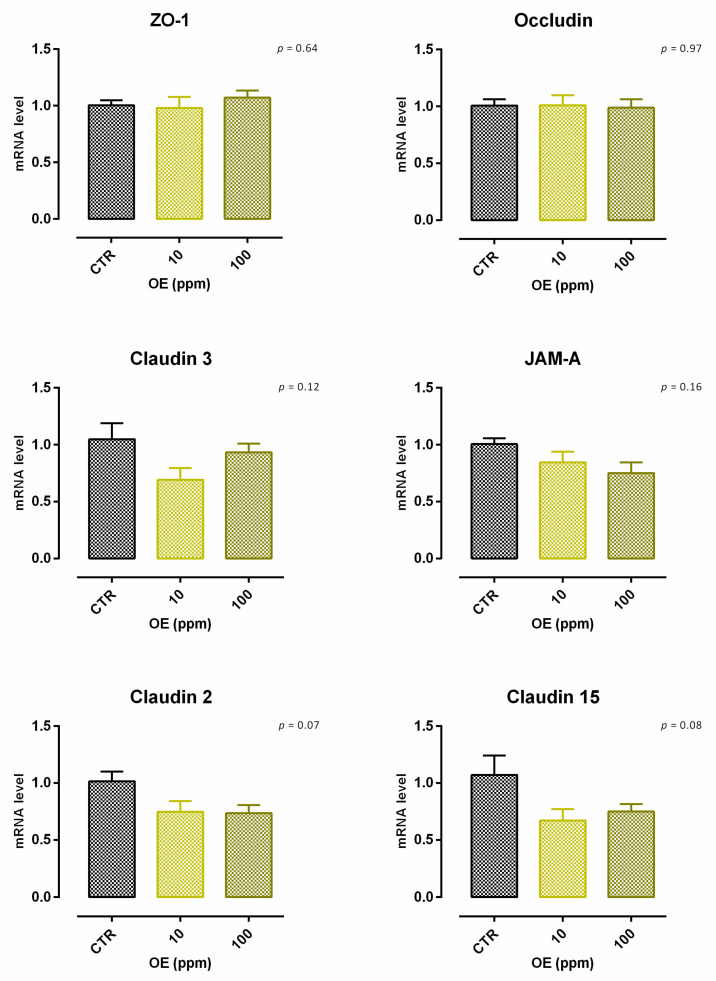
mRNA expression of tight junctions after 7 days of treatment with olive extract. Data are least square means (*n* = 6) ± SEM. Different letters indicate statistical significance with *p* < 0.05. ZO-1 = zonula occludens 1, JAM-A = Junctional adhesion molecules A.

A significant reduction (about 6%) in viability was reported with 10 ppm of OE after 24 h of treatment, and data were consistent after 7 days of treatment (Figure S5). Concerning TEER, 100 ppm of OE significantly increased the electrical resistance on days 2 and 5 of treatment, while a reduction was reported at the end of the experiment, with a tendency of increment (*p* = 0.06). Compared to the control, no variation was reported for cells with 10 ppm of OE (Figure 13). Results regarding gene expression are consistent with TEER, as reported in Figure 14. In fact, no significant variation in gene expression was reported. Also, a tendency for mRNA reduction was reported for claudin 2 and claudin 15 (*p* = 0.07 and *p* = 0.08, respectively).

#### 3.5.2. Antioxidant Activity

**Figure 15:**
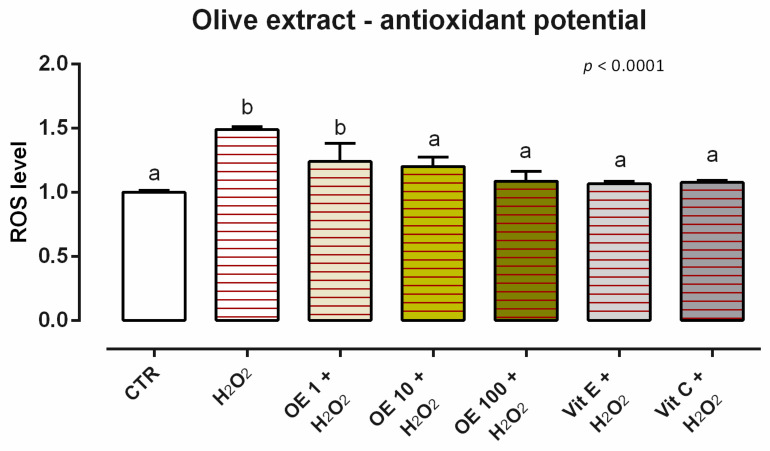
Effects of olive extract on intracellular ROS production: ROS level of Caco-2 cells treated with 1, 10, or 100 ppm of OE and challenged with 500 μM of hydrogen peroxide (H_2_O_2_) for 60 min. ROS production was assessed by CellROX^®^ deep red reagent assay and expressed as fluorescence intensity calculated relative to the control group. Values in the graph are means (*n* = 6) ± SEM. Different letters indicate statistical significance with *p* < 0.05. 500 μM of hydrogen peroxide (H_2_O_2_) was used as a positive control. Vitamin E (Vit E) and vitamin C (Vit C) were used as a standard of antioxidant potential.

After 24 h of incubation, OE induces ROS production for the concentrations tested (data not shown). When cells were challenged with H_2_O_2_ for 60 min (ROS-production challenge, positive control), the treatments with 10 and 100 ppm of OE allowed for a significant reduction of ROS concentration compared to the positive control, maintaining the same level of ROS as the unchallenged group and vitamins (internal controls) (Figure 15).

### 3.6. Pomegranate Extract

#### 3.6.1. Viability and Dose Response

**Figure 16:**
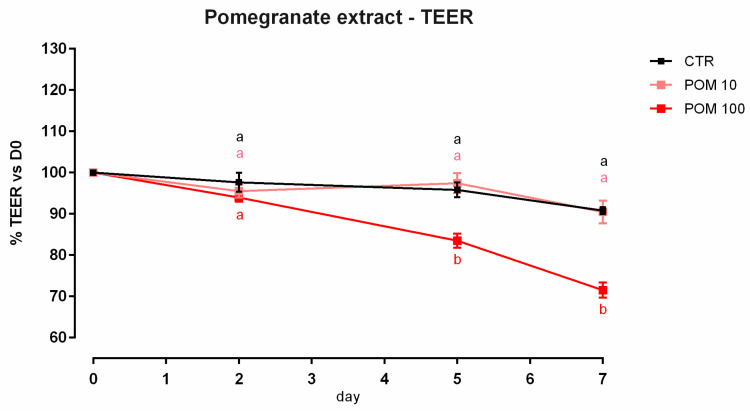
TEER of Caco-2 cells cultured with 10 or 100 ppm of pomegranate extract. Data in the graph are represented as means (*n* = 6) ± SEM. Different letters indicate statistical significance with *p* < 0.05. TEER = transepithelial electrical resistance (Ω·cm^2^).

**Figure 17:**
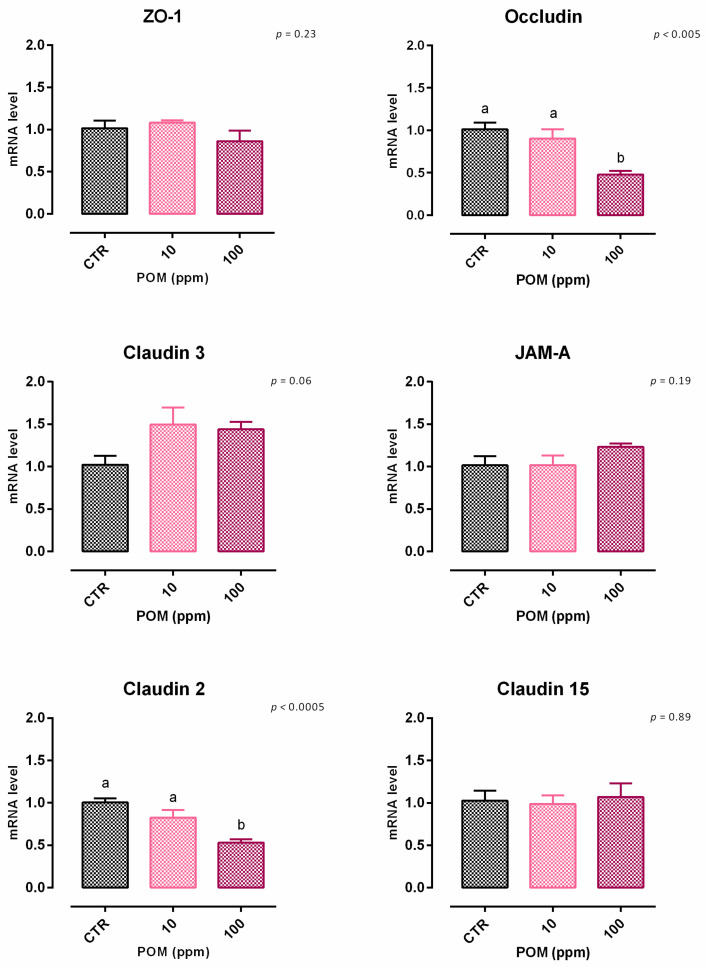
mRNA expression of tight junctions after 7 days of treatment with pomegranate extract. Data are least square means (*n* = 6) ± SEM. Different letters indicate statistical significance with *p* < 0.05. ZO-1 = zonula occludens 1, JAM-A = Junctional adhesion molecules A.

After 7 days, a significant reduction of viability was reported, starting from 100 ppm of inclusion (Figure S6). Concerning the dose response, 100 ppm of TTO negatively impacted the TEER after 5 days of the treatment, compared to the control, and the lower dosage of inclusion (Figure 16). Results regarding gene expression are reported in Figure 17. Compared to the control, the mRNA level of occludin was significantly reduced by the supplementation of 100 ppm of POM (*p* < 0.005), supporting the data obtained from TEER. Also, claudin 2 was negatively impacted by the inclusion of 100 ppm of POM (*p* < 0.0005). No differences were reported for ZO-1, JAM-A, and claudin 15, while a positive tendency was registered for claudin 3 (*p* = 0.06).

#### 3.6.2. Antioxidant Activity

**Figure 18:**
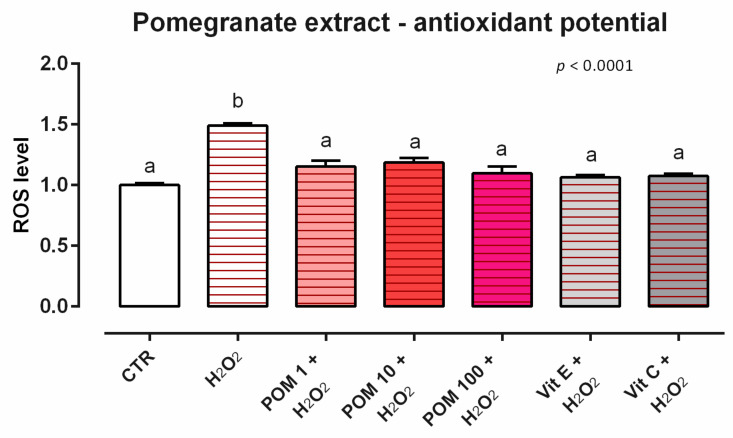
Effects of pomegranate extract on intracellular ROS production: ROS level of Caco-2 cells treated with 1, 10, or 100 ppm of POM and challenged with 500 μM of hydrogen peroxide (H_2_O_2_) for 60 min. ROS production was assessed by CellROX^®^ deep red reagent assay and expressed as fluorescence intensity calculated relative to the control group. VAlues in the graph are means (*n* = 6) ± SEM represented by vertical bars. Different letters indicate statistical significance with *p* < 0.05. 500 μM of hydrogen peroxide (H_2_O_2_) was used as a positive control. Vitamin E (Vit E) and vitamin C (Vit C) were used as a standard of antioxidant potential.

As concerns the ROS levels, 24 h of incubation with POM induced ROS production in Caco-2 cells at the tested concentrations (data not shown). When cells were subjected to a challenge with 500 µM of H_2_O_2_, the addition of POM positively impacted the ROS production, with a reduction starting from cells treated with the lower dose, compared to the positive control. The reduction of ROS obtained with POM was comparable to the one obtained from vitamins, which were used as internal controls (Figure 18).

### 3.7. Chestnut Extract

#### 3.7.1. Viability and Dose Response

**Figure 19:**
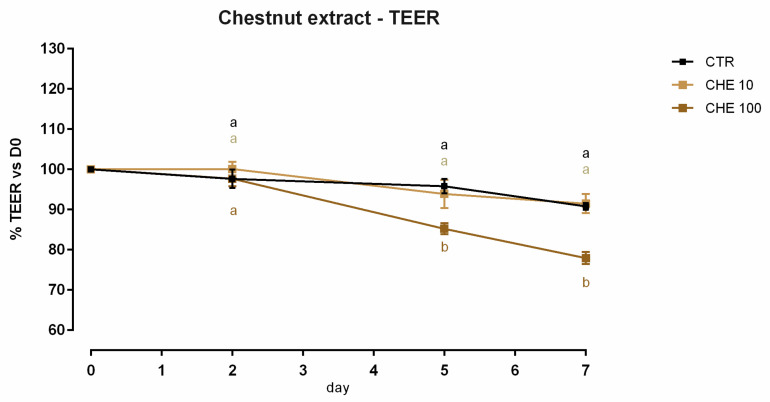
TEER of Caco-2 cells cultured with 10 or 100 ppm of chestnut extract. Data in the graph are represented as means (*n* = 6) ± SEM. Different letters indicate statistical significance with *p* < 0.05. TEER = transepithelial electrical resistance (Ω·cm^2^).

**Figure 20:**
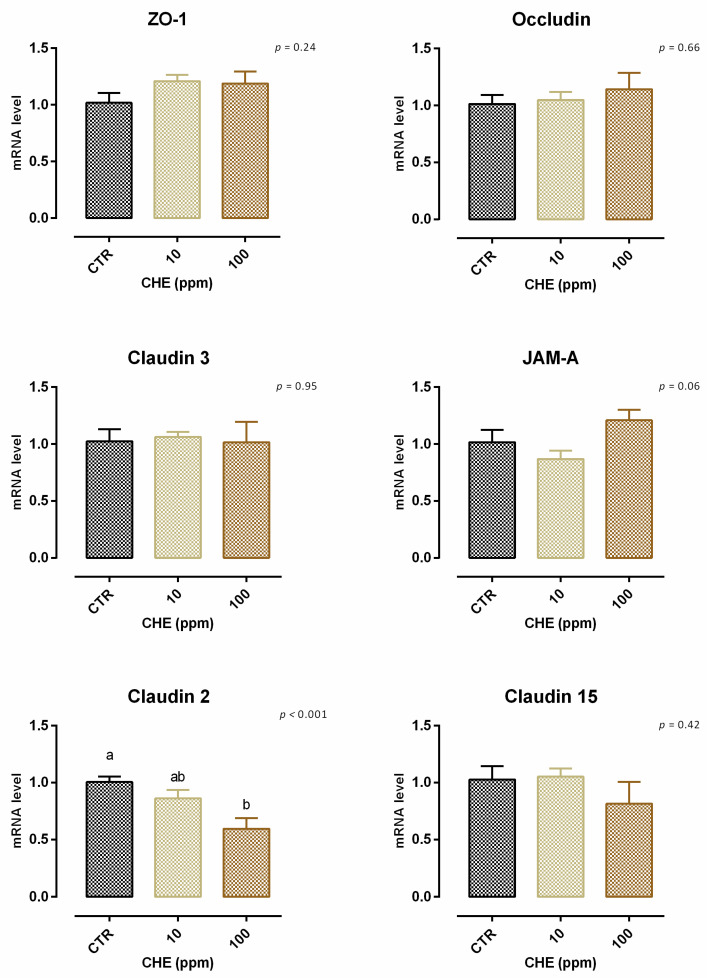
mRNA expression of tight junctions in Caco-2 cells after 7 days of treatment with chestnut extract. Data are least square means (*n* = 6) ± SEM. Different letters indicate statistical significance with *p* < 0.05. ZO-1 = zonula occludens 1, JAM-A = Junctional adhesion molecules A.

A slight but significant reduction (about 3%) of viability was reported with 1 ppm of CHE after 7 days of treatment; the reduction of viability for 10–100–200 ppm did not reach 10% (Figure S7). Concerning TEER, 100 ppm of CHE caused a significant decrease of the electrical resistance on days 5 and 7 of treatment, compared to the control and CHE 10. Compared to the control, no variation was reported for cells with 10 ppm of CHE (Figure 19). The results of gene expression are reported in Figure 20. No significant variation in gene expression was reported, with the exception of a reduction of claudin 2 (*p* < 0.001). A positive tendency was registered for JAM-A (*p* = 0.06).

#### 3.7.2. Antioxidant Activity

**Figure 21:**
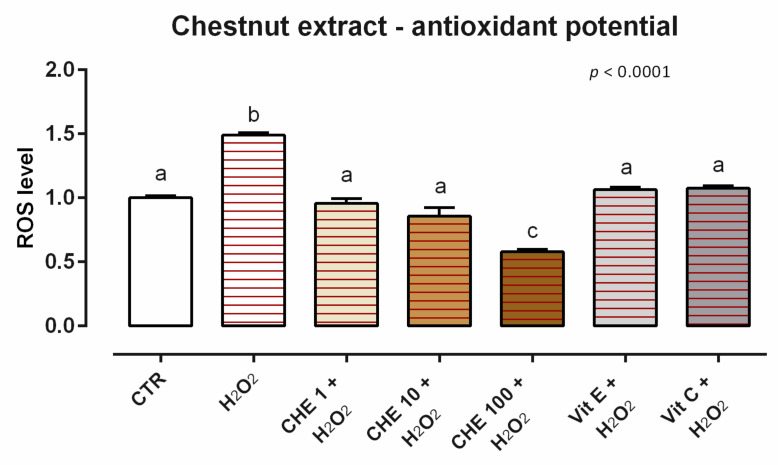
Effects of chestnut extract on intracellular ROS production: ROS level of Caco-2 cells treated with 1, 10, or 100 ppm of CHE and challenged with 500 μM of hydrogen peroxide (H_2_O_2_) for 60 min. ROS production was assessed by CellROX^®^ deep red reagent assay and expressed as fluorescence intensity calculated relative to the control group. Values in the graph are means (*n* = 6) ± SEM. Different letters indicate statistical significance with *p* < 0.05. 500 μM of hydrogen peroxide (H_2_O_2_) was used as a positive control. Vitamin E (Vit E) and vitamin C (Vit C) were used as a standard of antioxidant potential.

After 24 h of incubation, CHE induces ROS production for the concentrations tested (data not shown). When cells were challenged with H_2_O_2_ for 60 min (ROS-production challenge, positive control), the preventive treatments with CHE allowed for a significant reduction of ROS concentration compared to the positive control. In particular, 1 and 10 ppm allowed for a complete restorative effect, maintaining the same level of ROS as the unchallenged group and vitamins (internal controls), while 100 ppm promoted an antioxidative effect stronger than vitamins (Figure 21).

### 3.8. Thyme Essential Oil

#### 3.8.1. Viability and Dose Response

**Figure 22:**
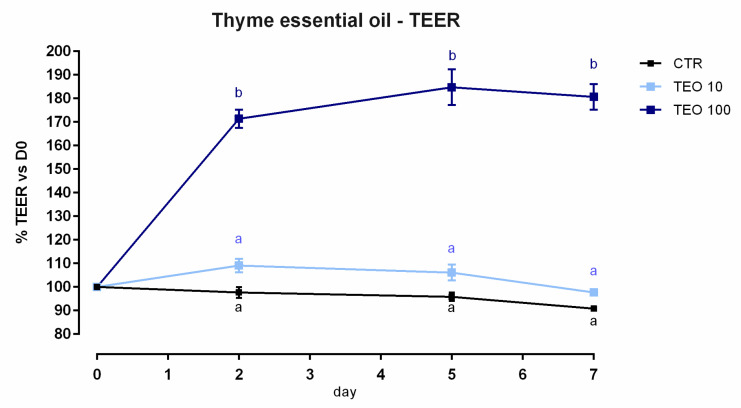
TEER of Caco-2 cells cultured with 10 or 100 ppm of thyme essential oil. Data in the graph are represented as means (*n* = 6) ± SEM. Different letters indicate statistical significance with *p* < 0.05. TEER = transepithelial electrical resistance (Ω·cm^2^).

**Figure 23:**
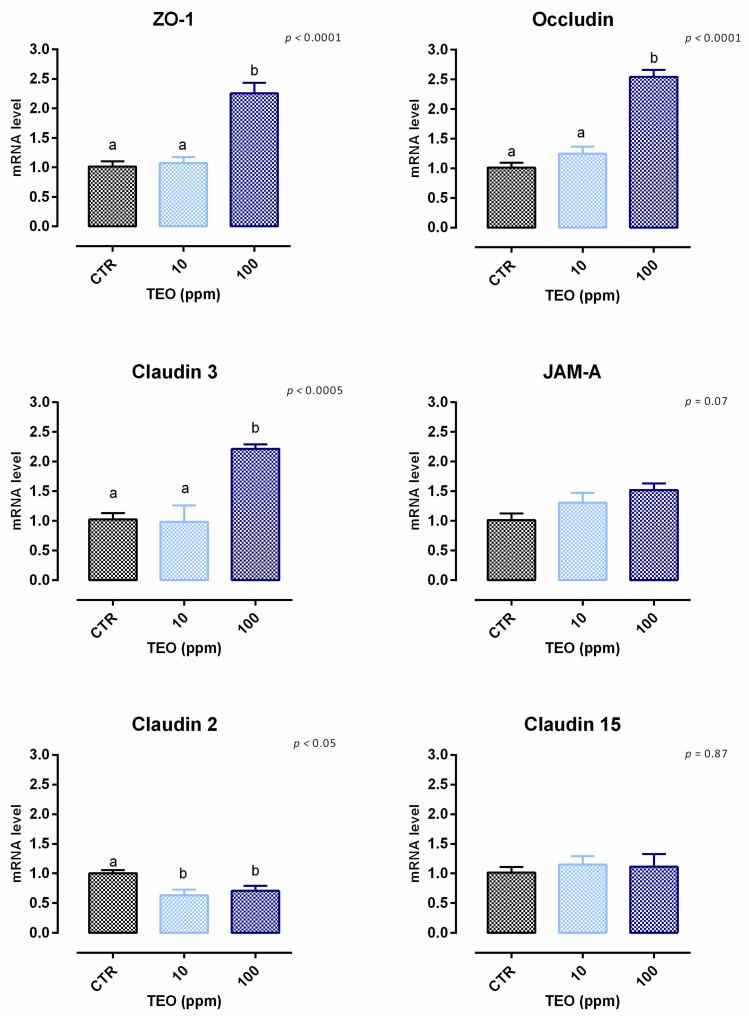
mRNA expression of tight junctions after 7 days of treatment with thyme essential oil. Data are least square means (*n* = 6) ± SEM. Different letters indicate statistical significance with *p* < 0.05. ZO-1 = zonula occludens 1, JAM-A = Junctional adhesion molecules A.

After 24 h, a slight but significant reduction of viability was reported starting from 1 ppm of inclusion (about 8%) (Figure S8). Regarding the dose response, 100 ppm of TEO allows a huge increment of TEER after 2 days of the treatment, and the improvement remained constant throughout the study (Figure 22). Results regarding gene expression are consistent with TEER, as reported in Figure 23. Compared to the control, mRNA levels of ZO-1, occludin, and claudin 3 were significantly improved by the treatment with 100 ppm of TEO (*p* < 0.0005). Also, JAM-A was tendentially increased by the treatment (*p* = 0.07). Conversely, claudin 2 mRNA levels were significantly reduced by adding TEO, with a similar reduction for the two inclusions (*p* < 0.05). No differences were reported for claudin 15.

#### 3.8.2. Antioxidant Activity

**Figure 24:**
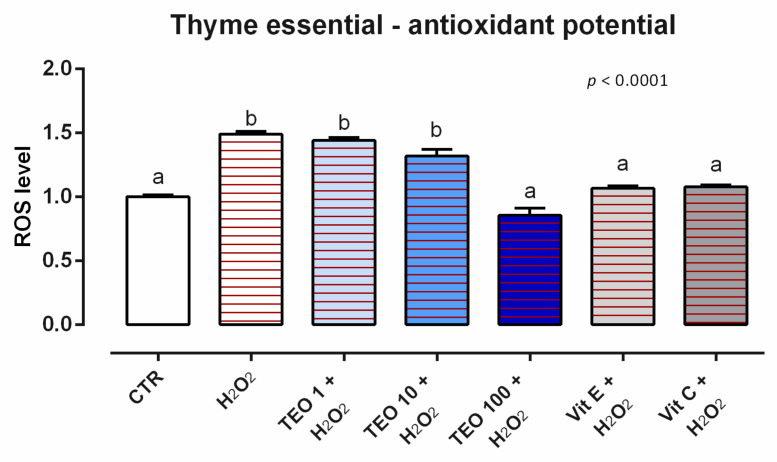
Effects of thyme essential oil on intracellular ROS production: ROS level of Caco-2 cells treated with 1, 10, or 100 ppm of TEO and challenged with 500 μM of hydrogen peroxide (H_2_O_2_) for 60 min. ROS production was assessed by CellROX^®^ deep red reagent assay and expressed as fluorescence intensity calculated relative to the control group. Values in the graph are means (*n* = 6) ± SEM. Different letters indicate statistical significance with *p* < 0.05. 500 μM of hydrogen peroxide (H_2_O_2_) was used as a positive control. Vitamin E (Vit E) and vitamin C (Vit C) were used as a standard of antioxidant potential.

After 24 h of incubation, no variation in ROS production was registered for any of the concentrations of TEO analyzed (data not shown). When cells were challenged with H_2_O_2_ for 60 min (ROS-production challenge, positive control), the treatments with 100 ppm of TEO significantly decreased the ROS concentration compared to the positive control, maintaining the same level of ROS as the unchallenged group and vitamins (internal controls) (Figure 24).

### 3.9. Capsicum Oleoresin

#### 3.9.1. Viability and Dose Response

**Figure 25:**
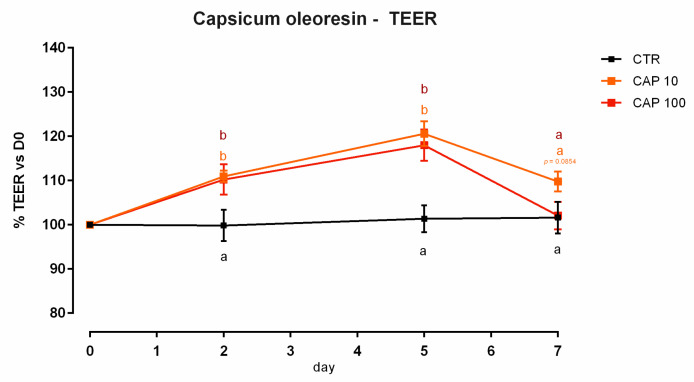
TEER of Caco-2 cells cultured with 10 or 100 ppm of capsicum oleoresin. Data in the graph are represented as means (*n* = 6) ± SEM. Different letters indicate statistical significance with *p* < 0.05 (a, b). TEER = transepithelial electrical resistance (Ω·cm^2^).

**Figure 26:**
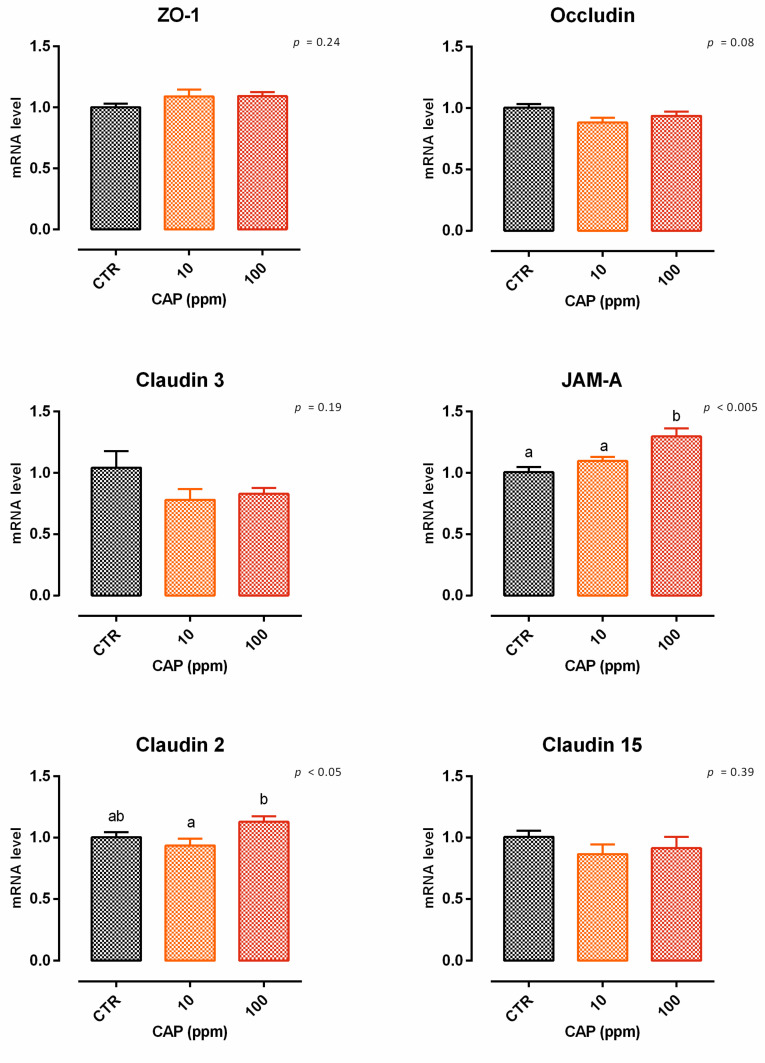
mRNA expression of tight junctions after 7 days of treatment with capsicum oleoresin. Data are least square means (*n* = 6) ± SEM. Different letters indicate statistical significance with *p* < 0.05. ZO-1 = zonula occludens 1, JAM-A = Junctional adhesion molecules A.

A reduction of nearly 5% of cell viability was reported after 24 h of treatment with 200 ppm of CAP (Figure S9). Concerning TEER, cells treated with 10 and 100 ppm showed an increment in TEER on days 2 and 5, compared to the control (Figure 25). Suddenly, at the end of the experiment, both the doses were unable to augment the TEER value compared to the control, with only a incremental tendency for cells with 10 ppm of CAP (*p* = 0.08 compared to the control). Compared to the control, the mRNA level of JAM-A was significantly improved by the addition of 100 ppm of capsicum (*p* < 0.005). The level of mRNA of claudin 2 was different between the treatments, but not different from the control. A tendency of reduction was reported for occludin (*p* = 0.08). No differences were reported for ZO-1, claudin 3, or claudin 15. The results of gene expression are summarized in Figure 26.

#### 3.9.2. Antioxidant Activity

**Figure 27:**
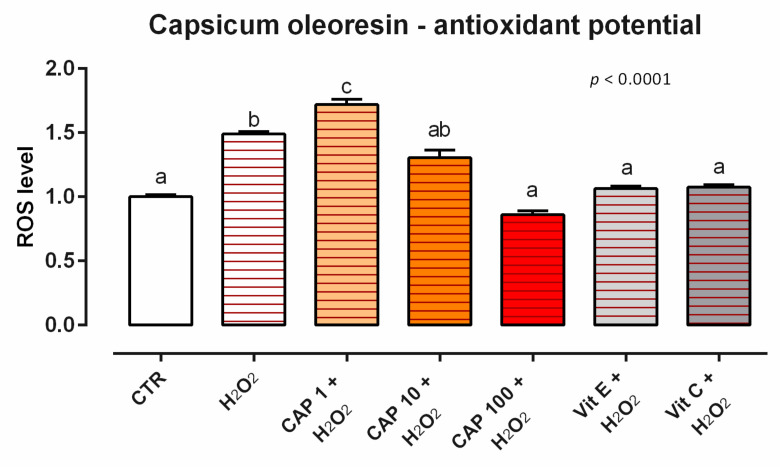
Effects of capsicum oleoresin on intracellular ROS production: ROS level of Caco-2 cells treated with 1, 10, or 100 ppm of CAP and challenged with 500 μM of hydrogen peroxide (H_2_O_2_) for 60 min. ROS production was assessed by CellROX^®^ deep red reagent assay and expressed as fluorescence intensity calculated relative to the control group. Values in the graph are means (*n* = 6) ± SEM. Different letters indicate statistical significance with *p* < 0.05. 500 μM of hydrogen peroxide (H_2_O_2_) was used as a positive control. Vitamin E (Vit E) and vitamin C (Vit C) were used as a standard of antioxidant potential.

As concerns the ROS levels, after 24 h of incubation with CAP, no difference in ROS production was described for the tested concentrations (data not shown). When cells were subjected to a challenge with 500 µM of H_2_O_2_, the addition of CAP positively impacted the ROS production, with an intermediate-to-total reduction of cells treated with 10 and 100 ppm of CAP. In particular, an intermediate reduction of ROS was observable with 10 ppm, while the preventive treatment with CAP 100 ppm allowed for a complete restoration of the redox status, with ROS value comparable to control and vitamins (Figure 27).

## 4. Discussion

Intestinal health is closely connected with the mucosa and immune system, whose importance in human and animal wellbeing is currently well established. Particularly, in addition to the classical functions of food digestion, absorption, and metabolism, the intestine also plays a key role as a physical and immunological barrier. The complex interplay between healthy mucosa and the external environment contributes to various vital functions such as supporting digestion, acting as barrier against pathogens, supporting the host immune system, maintaining redox status, and supporting the gut-brain axis [23]. Botanicals are one of the most popular complementary and alternative non-conventional medications, which continue to grow in popularity, even in animal production, due to the desire for a reduction of the use of antibiotics [24,25] and due to the therapeutic potential of natural substances [26]. Phenolic components are widely used in animal and human nutrition for their countless beneficial properties, mainly as antioxidants and anti-inflammatories [27,28,29,30,31,32,33,34]. The present study was conducted to investigate the effects of phenol-rich botanicals from ginger, tea tree, grape seed, green tea, olive, pomegranate, chestnut, thyme, and capsicum on intestinal integrity and oxidative stress in Caco-2 cells. All these substances are approved as feed additives and are rich in phenolic compounds, one of the major groups of botanical components. The results obtained offer an overview of the potential use of these botanicals as dietary supplements for their antioxidant capacities. Moreover, the obtained data provides an insight into the capacities of these phenol-rich botanicals to modulate the integrity of the intestinal barrier, which is poorly described in literature. Clearly, the wide variety of phenolic components enables a different response in the organism. To the best of our knowledge, this is the first approach to assessing the role of these natural substances in vitro on enterocytes, as there have been few studies of this type conducted on Caco-2 cells.

The production of ROS is a key event in the progression of many inflammatory disorders, including those involving the gastrointestinal tract. Uncontrolled and persistent oxidative stress with overproduction of ROS, and/or inadequate removal of ROS by antioxidant systems, can cause apoptosis and tissue injury [35,36]. An increase of ROS production following a GSH depletion represents a crucial event that irreversibly activates apoptotic signaling [37].

All of the tested botanicals allowed for the depletion of the levels of H_2_O_2_-induced ROS in Caco-2 cells, helping maintain the redox status under physiological conditions. Flavonoids might exert direct antioxidant effects by scavenging reactive species, as well as by modulating antioxidant enzymes [38]. This reflects the strong antioxidant potential shown with the addition of GEO, GSE, and TTO. Numerous studies have reported that GEO has a strong antioxidant activity due to the increased activity of antioxidant enzymes, such as superoxide dismutase and catalase [39,40,41]. Ginger’s antioxidant potential is probably driven by 6-shogaol and 6-gingerol. Indeed, 6-shogaol, one of the most abundant phenols of GEO, was able to reduce the levels of malondialdehyde and increase glutathione in rats, via activation of the Nrf2 (nuclear factor erythroid 2-related factor 2)-signaling pathway [42]. Also, 6-gingerol is able to inhibit the formation of ROS in intestinal mucosa injury [43]. The results obtained from GSE found support in the study performed by Pinent et al. [44], which reported the ability of GSE to prevent oxidative stress in pre-treated Caco-2 cells. Proanthocyanidins, highly represented in green tea, as well as in grape seeds [45,46], have a strong antioxidant activity [47]. GTE, OE, and CAP exerted their antioxidant potential starting from 10 ppm of inclusion, probably due to the different flavonoids in their composition. The potent antioxidant activity of oleuropein and capsaicin, the principal phenolic compounds of OE and CAP, respectively, is mainly due to the presence of hydroxyl groups that could donate hydrogen to prevent oxidation [48]. Moreover, oleuropein strongly inhibits copper sulphate-induced oxidation and increases the inducible nitric oxide synthase (iNOS) expression in the cell [49].

Tannins, highly represented in pomegranate and chestnut extract, are considered anti-nutritional substances [50], but are also known as a strong antioxidant. In fact, tannins have the ability to chelate metal ions, retarding oxidation, and inhibit lipid peroxidation via the inhibition of cyclooxygenase activity [51]. Therefore, POM and CHE, whose composition rich in tannins produced a reduction of TEER, appeared highly useful against ROS. Also, the key active compounds in pomegranate fruit, a group of hydrolyzable ellagitannins generically called punicosides, can reduce macrophage oxidative stress, free radicals, and lipid peroxidation, thanks to the inhibition of cyclooxygenase and pro-inflammatory cytokines [52]. The EO obtained from thyme appears less effective than other botanicals in scavenging free radicals, as the antioxidant activity is mainly expressed at the highest dose. The effects are probably driven by thymol, known for its ability to scavenge free radicals, enhance the endogenous enzymatic and non-enzymatic antioxidants, and chelate metal ions [53].

Intestinal oxidative stress plays a pivotal role in the primary stage of intestinal injury, leading to intestinal barrier dysfunction, thus triggering inflammation and immune imbalance [54]. Despite the growing interest in the use of botanicals in animal and human nutrition, it is interesting to note that few studies have been conducted to evaluate the role of these natural extracts on intestinal barrier functionality. Even though the present study was restricted to the investigation of only some characteristics of intestinal barrier function, it is remarkable how the selected botanicals appear able to influence the monolayer of enterocytes, probably due to their antioxidant capacity. While GEO, TTO, GSE, GTE, OE, TEO, and CAP conferred enhanced resistance to polarized Caco-2, despite individual differences, the remaining extracts showed an impairment of TEER values. Considering that the measurement of TEER reflects the ionic conductance, as well as the pore size of the tight junctions (TJs) [55], these variations could be related to a shift in the TJ’s composition. Indeed, the differences between the TEER patterns are mostly confirmed by the TJs gene expression. In literature, some flavonoid-rich extracts are described as being able to produce a change in the composition of TJs [56,57,58].

Regarding the barrier integrity, ginger and thyme produced a similar trend, with a large improvement of electrical resistance and TJs gene expression, especially at 100 ppm. In addition, both GEO and TEO allowed a reduction of mRNA levels of claudin 2 and claudin 15, which are normally recognized as “leaky gut” markers [59,60]. Over the last few years, the active ingredients of ginger and thyme, both widely used as flavorings and food ingredients, have been studied as natural antimicrobial agents [61,62]. 6-shogaol was found to exert barrier-protective effects by preventing TJs modifications after inflammatory challenge in vitro and in vivo [63,64]. Moreover, thymol is well known for its positive modulation of intestinal barrier function [15,65]. Tea tree, grape seeds, and green tea showed an interesting increase of resistance at both doses, partially supported by gene expression of TJs to a lesser extent than GEO and TEO. Some in vitro studies have associated proanthocyanidins with augmented TEER and reduced permeability in situations of barrier dysfunction, reinforcing data obtained from GSE and GTE [66,67]. Also, terpinene-4-ol (primary constituent of TTO) was reported to positively impact impaired intestinal barrier function, alleviating the negative effects correlated with lipopolysaccharide (LPS) and dextran sulfate sodium (DSS) [68]. Capsicum and olive appear advantageous when applied for shorter periods, as both allowed an increase of TEER over the initial period of supplementation. Capsaicin induces the reversible opening of TJs associated with a Ca^2+^ influx, which has been associated with a reduction of occludin and actin alteration [69,70]. In fact, capsicum extract is under investigation for its ability to increase energy metabolism in intestinal epithelial cells [71], to modulate growth performance, nutrient utilization, antioxidant status, and immune function in broilers [72], and act as a modulator of obesity in humans [73]. This knowledge, as well as the results obtained in this paper, suggests a positive activity of capsicum, although a high concentration should be avoided. On the other hand, it appears clear that the highest dose of pomegranate and chestnut triggers a loss of epithelial tightness. Nevertheless, claudin 2 was always reduced by the treatments, suggesting a positive modulation of intestinal health despite the reduction of TEER. In fact, claudin 2 expression is dysregulated in cancer and inflammation, with a positive correlation between disease stage and claudin 2 abundance [74]. In some studies, tannins induced structural disruption of the intestinal TJs [75,76]. This partially explains the reduction of TEER registered in our results. For pomegranate and chestnut, low to moderate concentrations can exert a positive modulation of intestinal functions, avoiding the undesirable effects associated with a surplus of tannins [77].

These results provide an initial overview of the effects of phenol-rich botanicals on the intestinal barrier, which is probably heavily influenced by antioxidant activity. In fact, the combined data from antioxidant assays and barrier integrity suggest that the tested phenol-rich botanicals have a dual potential: besides their well-known capacity to scavenge free radicals and modulate enzyme activity, they also reveal an unexplored capacity to modulate barrier integrity. Thus, phenol-rich botanicals have the potential to sustain intestinal health by regulating the expression of TJs and by making the intestinal epithelium more resistant to oxidative and inflammatory challenges, which might come from various external stimuli. In a positive feedback manner, antioxidant ability allows for the controlling of oxidative stress, maintaining the equilibrium of the redox state in epithelial cells, with a consequent improvement of barrier integrity.

Nevertheless, this is just a preliminary screening of the potential of phenol-rich botanicals in nutrition. More data are needed in terms of the acceptability and palatability of these extracts as dietary supplements in feed and food, as well as data on their additional properties. It is realistic to hypothesize that the ability of phenol-rich botanicals to improve intestinal barrier integrity plays a role in other intestinal functions, such as reducing pathogen proliferation and translocation, alleviating inflammation, reducing stress, and changing gut-brain interactions.

## 5. Conclusions

Phenol-rich botanicals have different chemical and biological properties with a wide range of molecular targets, showing the potential to ameliorate intestinal oxidative status and barrier function in vitro. The results suggest the potential use of these extracts as dietary supplements to improve intestinal barrier functionality. Ginger, tea tree, grape seeds, green tea, olive, pomegranate, chestnut, thyme, and capsicum were all sources of phenolic compounds, which demonstrate the ability to modulate intestinal oxidative stress and mucosal integrity. The beneficial effects of phenol-rich botanicals on animal and human health could be related to the oxidative homeostasis of the intestinal cells and the integrity of the intestinal barrier, which is maintained by the antioxidant function of phenolic compounds. Thus, a chemical characterization is important in order to recognize the major components of botanicals and their ratio, which should facilitate an understanding of the main drivers of their desired effects.

## Figures and Tables

**Table 1:** Primers used for gene expression analysis.

Gene	Primer Sequence (F and R) 5′→3′	Product Length (bp)	Accession N.	Reference
ZO-1	F: CGGGACTGTTGGTATTGGCTAGA	184	NM_001301025.3	[18]
	R: GGCCAGGGCCATAGTAAAGTTTG			
OCCL	F: TCCTATAAATCCACGCCGGTTC	105	NM_001205254.2	[18]
	R: CTCAAAGTTACCACCGCTGCTG			
CLDN-2	F: ATTGTGACAGCAGTTGGCTT	86	NM_001171092.1	[19]
	R: CTATAGATGTCACACTGGGTGATG			
CLDN-3	F: ACATCATCACGTCGCAGAACATC	103	NM_001306.4	[18]
	R: AGTGCCAGCAGCGAGTCGTA			
CLDN-15	F: TCCTATAAATCCACGCCGGTTC	155	NM_001185080.2	[20]
	R: CTCAAAGTTACCACCGCTGCTG			
JAM-A	F: CAGAGGTGATTCATGGCTCTGTG	96	NM_001382727	[18]
	R: TTCCAGGCTGGCAATAACTGAC			
RPLP0	F: GCAATGTTGCCAGTGTCTG	142	NM_001002.3	[21]
	R: GCCTTGACCTTTTCAGCAA			
GAPDH	F: TGCACCACCAACTGCTTAGC	87	NM_02046	[22]
	R: GGCATGGACTGTGGTCATGAG			

F = forward; R = reverse; ZO-1 = zonula occludens-1; OCCL = occludin; CLDN-2 = claudin 2; CLDN-3 = claudin 3; CLDN-15 = claudin 15; JAM-A = Junctional adhesion molecule A; RPLP0 = Ribosomal protein lateral stalk subunit P0; GAPDH = Glyceraldehyde 3-phosphate dehydrogenase.

## Data Availability

The data presented in this study are available on request from the corresponding author.

## Supplementary Materials

The following are available online at https://www.mdpi.com/article/10.3390/ani12172188/s1, Figure S1: Viability of Caco-2 cells treated for 24 h and 7 days with incremental doses of ginger essential oil, Figure S2: Viability of Caco-2 cells treated for 24 h and 7 days with incremental doses of tea tree oil, Figure S3: Viability of Caco-2 cells treated for 24 h and 7 days with incremental doses of grape seed extract, Figure S4: Viability of Caco-2 cells treated for 24 h and 7 days with incremental doses of green tea extract, Figure S5: Viability of Caco-2 cells treated for 24 h and 7 days with incremental doses of olive extract, Figure S6: Viability of Caco-2 cells treated for 24 h and 7 days with incremental doses of pomegranate extract, Figure S7: Viability of Caco-2 cells treated for 24 h and 7 days with incremental doses of chestnut extract, Figure S8: Viability of Caco-2 cells treated for 24 h and 7 days with incremental doses of thyme essential oil, Figure S9: Viability of Caco-2 cells treated for 24 h and 7 days with incremental doses of capsicum oleoresin.
