# Supplementary material for: Phenol-Rich Botanicals Modulate Oxidative Stress and Epithelial Integrity in Intestinal Epithelial Cells
Source: Animals (Basel). 2022 Aug 25;12(17):2188. doi: 10.3390/ani12172188 (PMC9454507; doi:10.3390/ani12172188)
Supplement: Supplementary file 1 [file animals-12-02188-s001.zip › animals-1876826-supplementary.pdf]

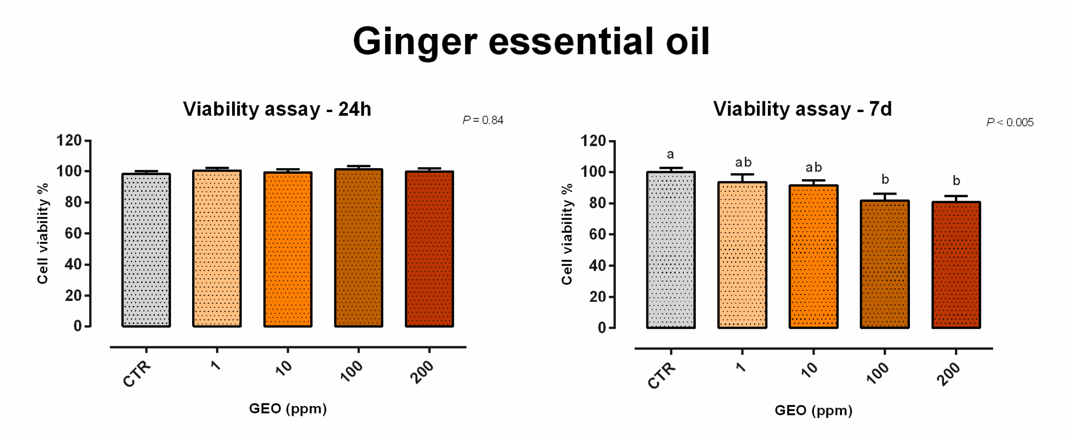

Figure S1. Viability of Caco-2 cells treated for 24 hours and 7 days with incremental doses of ginger essential oil. Data in the graph are means (n = 6) ± SEM represented by vertical bars. Means with different letters indicate statistical significance with $p < 0.05$ (a, b); means with at least one common letter are not significantly different (ab).

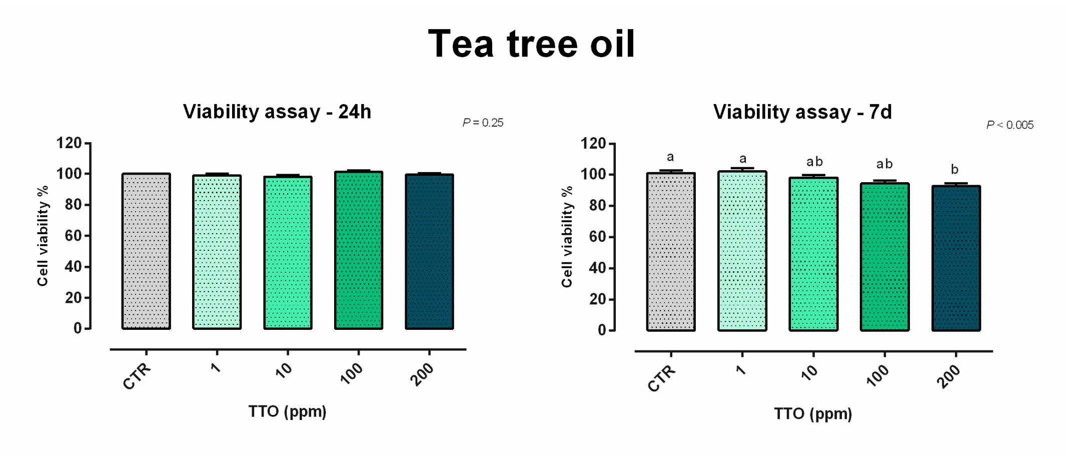

Figure S2. Viability of Caco-2 cells treated for 24 hours and 7 days with incremental doses of tea tree oil. Data in the graph are means (n = 6) ± SEM represented by vertical bars. Means with different letters indicate statistical significance with $p < 0.05$ (a, b); means with at least one common letter are not significantly different (ab).

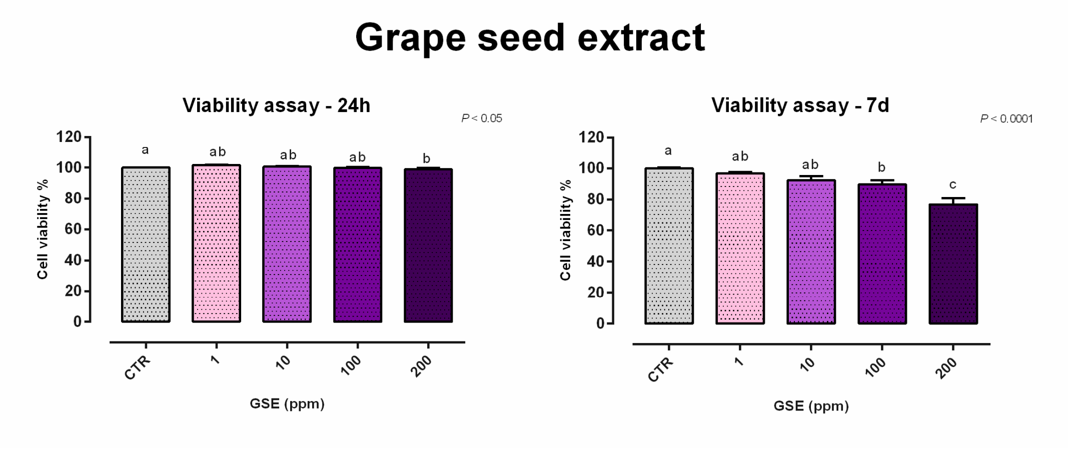

Figure S3. Viability of Caco-2 cells treated for 24 hours and 7 days with incremental doses of grape seed extract. Data in the graph are means (n = 6) ± SEM represented by vertical bars. Means with different letters indicate statistical significance with $p < 0.05$ (a, b); means with at least one common letter are not significantly different (ab).

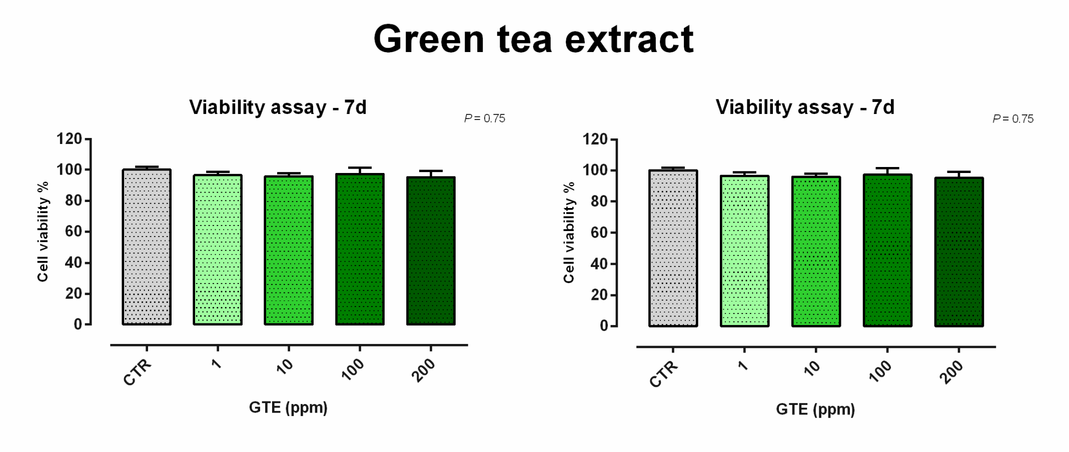

Figure S4. Viability of Caco-2 cells treated for 24 hours and 7 days with incremental doses of green tea extract. Data in the graph are means (n = 6) ± SEM represented by vertical bars. Means with different letters indicate statistical significance with $p < 0.05$ (a, b); means with at least one common letter are not significantly different (ab).

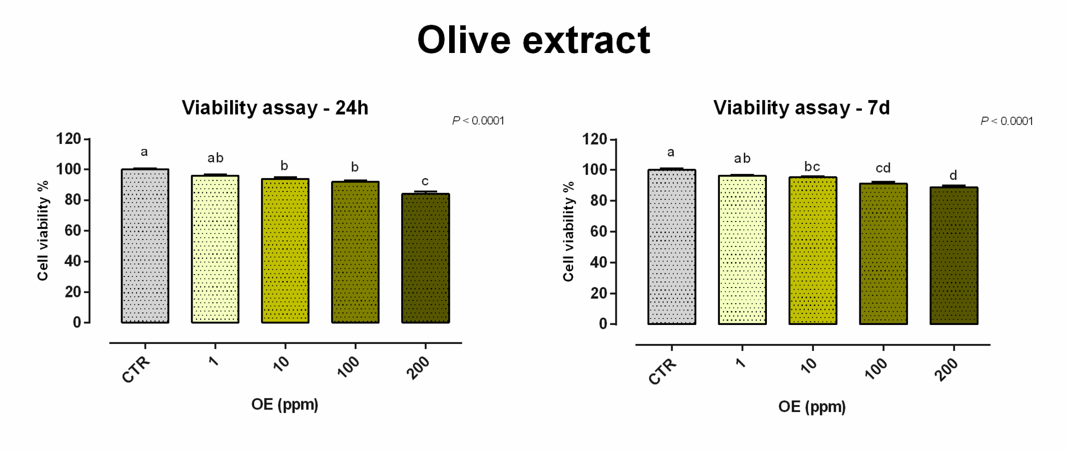

Figure S5. Viability of Caco-2 cells treated for 24 hours and 7 days with incremental doses of olive extract. Data in the graph are means (n = 6) ± SEM represented by vertical bars. Means with different letters indicate statistical significance with $p < 0.05$ (a, b); means with at least one common letter are not significantly different (ab).

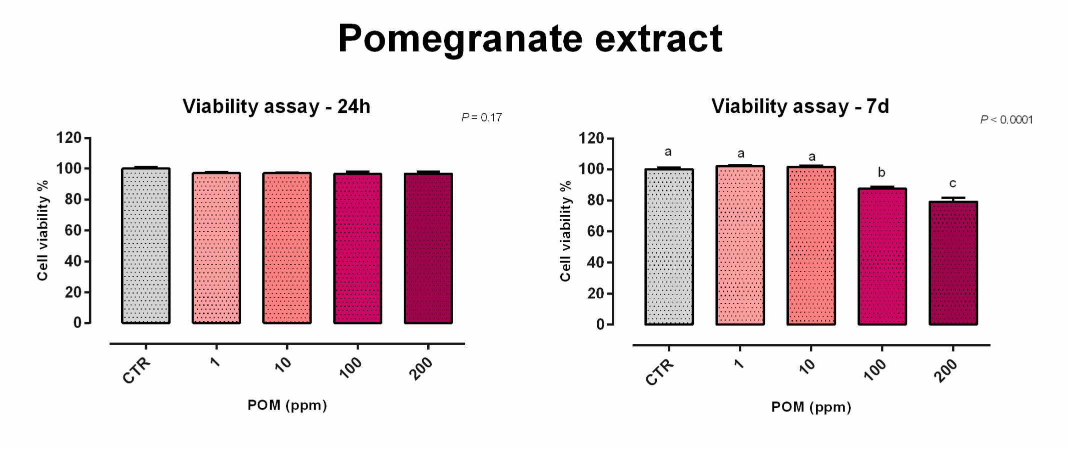

Figure S6. Viability of Caco-2 cells treated for 24 hours and 7 days with incremental doses of pomegranate extract. Data in the graph are means (n = 6) ± SEM represented by vertical bars. Means with different letters indicate statistical significance with $p < 0.05$ (a, b); means with at least one common letter are not significantly different (ab).

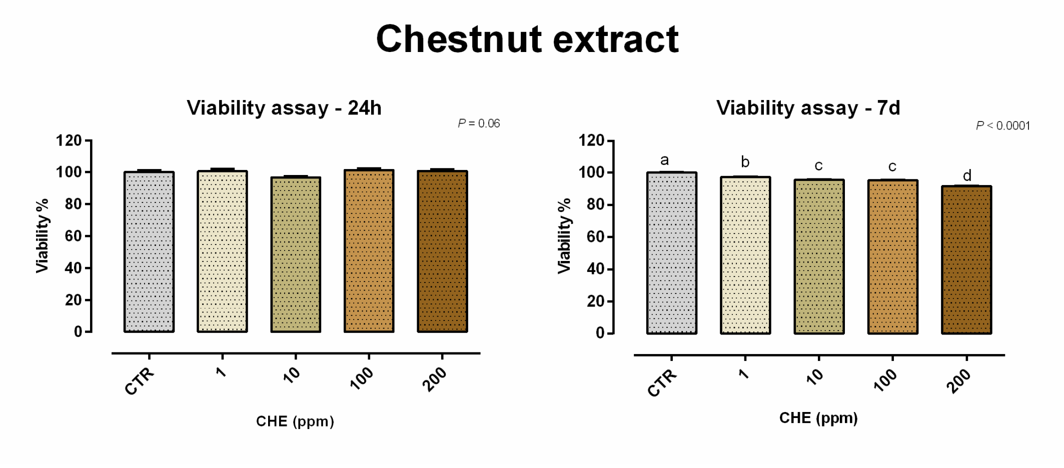

Figure S7. Viability of Caco-2 cells treated for 24 hours and 7 days with incremental doses of chestnut extract. Data in the graph are means (n = 6) ± SEM represented by vertical bars. Means with different letters indicate statistical significance with $p < 0.05$ (a, b); means with at least one common letter are not significantly different (ab).

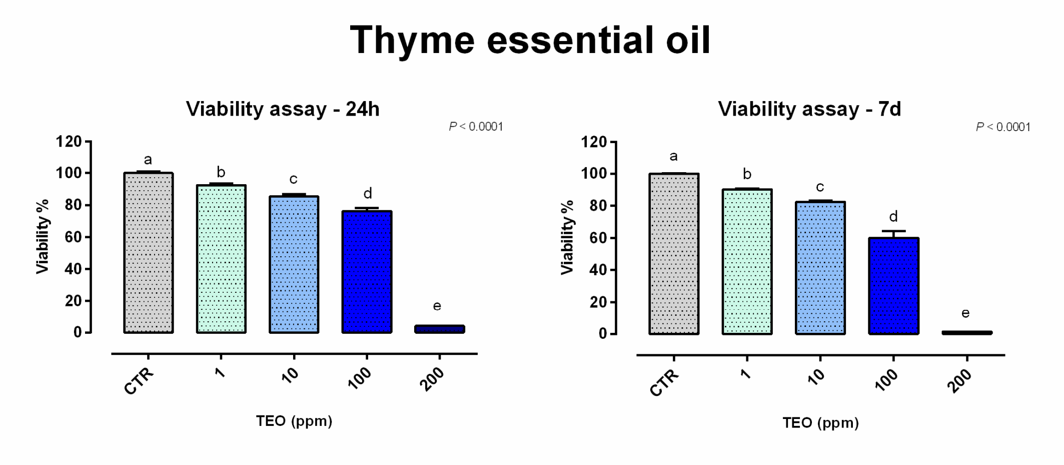

Figure S8. Viability of Caco-2 cells treated for 24 hours and 7 days with incremental doses of thyme essential oil. Data in the graph are means (n = 6) ± SEM represented by vertical bars. Means with different letters indicate statistical significance with $p < 0.05$ (a, b); means with at least one common letter are not significantly different (ab).

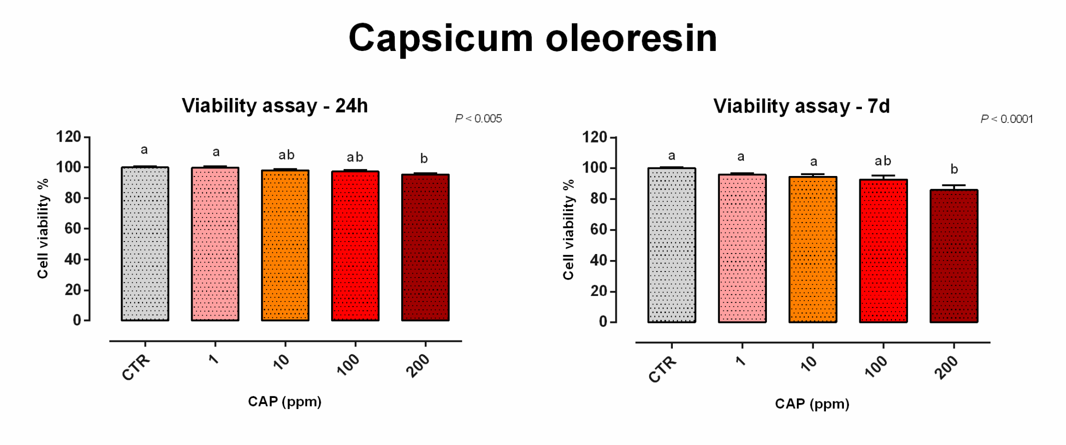

Figure S9. Viability of Caco-2 cells treated for 24 hours and 7 days with incremental doses of capsicum oleoresin. Data in the graph are means (n = 6) ± SEM represented by vertical bars. Means with different letters indicate statistical significance with $p < 0.05$ (a, b); means with at least one common letter are not significantly different (ab).
